# Effects of Vitamin D Supplementation and a Cafeteria Diet on Various Parameters in the Next Generation of Rats with Metabolic Syndrome

**DOI:** 10.3390/nu16213781

**Published:** 2024-11-04

**Authors:** İsmail Caner Yavuz, Betül Çiçek

**Affiliations:** 1 Department of Health Care Services, Vocational Health College, Süleyman Demirel University, Isparta 32260, Türkiye; 2 Department of Nutrition and Dietetics, Faculty of Health Sciences, Erciyes University, Kayseri 38280, Türkiye; bcicek@erciyes.edu.tr

**Keywords:** metabolic syndrome, cafeteria diet, vitamin D, fasting serum glucose, obesity

## Abstract

Background/Objectives: Metabolic Syndrome (MetS) is an increasingly widespread public health problem worldwide. MetS is associated with a cafeteria diet characterized by high fat and high simple carbohydrates. A cafeteria diet significantly affects serum glucose, creatine, urea, triglyceride, cholesterol and MetS parameters such as ALT, AST and ALP. Due to its epigenetic effects, vitamin D is important in controlling MetS parameters and minimizing MetS findings in subsequent generations. Methods: In this study, the effect of weekly 0.3 mL (1.000 IU/week) vitamin D intervention on MetS parameters was investigated in parental rats developing high-fructose MetS and their offspring. Offspring of MetS rats receiving and not receiving vitamin D supplementation were divided into four different groups and exposed to a cafeteria diet and vitamin D supplementation for eight weeks. Results: It was shown that parental rats in the intervention group had lower serum urea, glucose, creatine, total cholesterol, ALP, AST and ALT levels (*p* < 0.05). Serum urea, glucose, creatine, ALT, AST, ALP, triglyceride, total cholesterol levels and body weights were lower and HDL levels were higher in the offspring (*p* < 0.05). However, initial serum ALT and AST values were higher in the offspring of MetS parent rats receiving vitamin D supplementation and in the offspring of rats not receiving supplementation than in the offspring of supplemented parents. Conclusions: In conclusion, it was found that vitamin D supplementation improved MetS parameters in parent rats, positively affected MetS parameters in offspring rats despite an inadequate diet, and positively affected some MetS parameters by affecting epigenetic pathways in offspring born to MetS mothers.

## 1. Introduction

MetS is a complex disease that is increasingly prevalent and is characterized by elevated fasting blood glucose levels, atherogenic dyslipidemia, liver enzyme deterioration and obesity, and which causes morbidity and mortality [1,2]. The main criteria for the diagnosis of MetS are abnormal changes in serum-impaired fasting glucose, liver enzymes (ALT, AST, ALP), inflammation, creatine, urea, total cholesterol, HDL cholesterol and body weight [3]. Diet is one of the triggers of metabolic syndrome. The components and nutrients in an individual’s food consumption constitute an important risk factor for the development of MetS in the long term. The main risk factors are advances in technology, socioeconomic life and the so-called “cafeteria diet”, which has recently been positively correlated with the development of MetS [4,5]. This diet, characterized by high fat and high simple carbohydrate ratios, primarily increases circulating levels of free fatty acids, causes adipose tissue dysfunction, accelerates the development of obesity, increases inflammatory markers, disrupts glucose and lipid homeostasis and leads to the development of MetS [6]. In a study conducted with rats by Zhuhua et al. [7], it was found that rats fed a high fat and high fructose diet had higher blood glucose, hyperinsulinemia, fat mass, dyslipidemia and glucose intolerance. In another study, conducted by Lee et al. [8] with 7568 patients, a positive correlation was found between serum ALT levels and the prevalence of MetS. The greatest effect of a cafeteria diet on MetS development is on the liver, pancreas, kidneys and endocrine system. In particular, blood lipid profiles and inflammatory markers disrupted by high fat and/or high carbohydrate diets may trigger the development of autoimmune diseases [9]. One of the healthiest supplements to minimize health problems caused by MetS, reduce symptoms and manage the disease is vitamin D supplementation. Vitamin D has many different functions in the human body. These include endocrine, autocrine and paracrine functions and hormonal activity. In addition to all these activities, vitamin D has other pleiotropic functions such as anti-inflammatory, anti-apoptotic and anti-fibrotic effects, and protective effects against cardiovascular and kidney diseases, diabetes or cancer [10,11]. In addition, impaired serum vitamin D levels can cause and/or worsen glucose intolerance, and vitamin D deficiency is associated with obesity by causing an increase in adipose tissue; hypertriglyceridemia and vitamin D levels show an inverse relationship [12]. In MetS cases that develop due to modifiable causes such as nutrition, the literature data prove that vitamin D intake has an ameliorative effect on MetS parameters such as insulin resistance, fasting glucose levels, body weight and serum lipid profiles by affecting histone modification, DNA methylation and miRNA expression [13,14]. However, the impact of this on MetS parameters in subsequent generations is not fully known. In this experimental animal study, we aimed to investigate the effects of vitamin D supplementation on various MetS parameters in MetS-induced parental rats and the effects of vitamin D supplementation on various MetS parameters in offspring born from MetS-induced parental rats in a cafeteria-style feeding model and to contribute to the literature.

## 2. Materials and Methods

### 2.1. Study Design

This experimental animal study was designed to investigate the relationship between cafeteria diet and MetS development in rats, to determine the effect of MetS on the next generation and to evaluate the ameliorative effect of vitamin D on MetS parameters. This study design provided a high level of compliance with the purpose of the study, considering the easy availability of the sample, the easy development of MetS in rats and the rapid acquisition of findings.

### 2.2. Inclusion Criteria

The study included *n* = 12 female Wistar Albino rats between 200 and 250 g and *n* = 4 male parent rats between 300 and 350 g, aged 16–24 weeks, raised under standard laboratory conditions, and *n* = 24 randomly selected offspring of the matings of these parent rats, upon reaching adulthood, regardless of gender.

### 2.3. Exclusion Criteria

Wistar Albino rats that were unhealthy and/or had low body weight and did not reach adulthood were excluded from the study.

### 2.4. Sample Size

The power analysis of the study was performed with the GPower 3.1.9.2. (Universitaet Kiel, Kiel, Germany) program. Since the study was designed for independent groups and repeated measures, “F test” was selected as the test family, and “ANOVA: Repeated measures, within-between interaction” was selected as the analysis tool. In the calculation made using the biochemical values obtained in the pilot study, the eta-squared value was found to be 0.24. The margin of error in the power analysis was taken as 5% and the power value as 95%. The effect size was calculated as d = 0.561 and the total sample size was calculated as N = 16 (n = 12 females and n = 4 males) for two adult groups, four juvenile groups, and two measurements as N = 24. The study was completed for n = 6 rats in each group.

### 2.5. Study Plan

**Figure 1:**
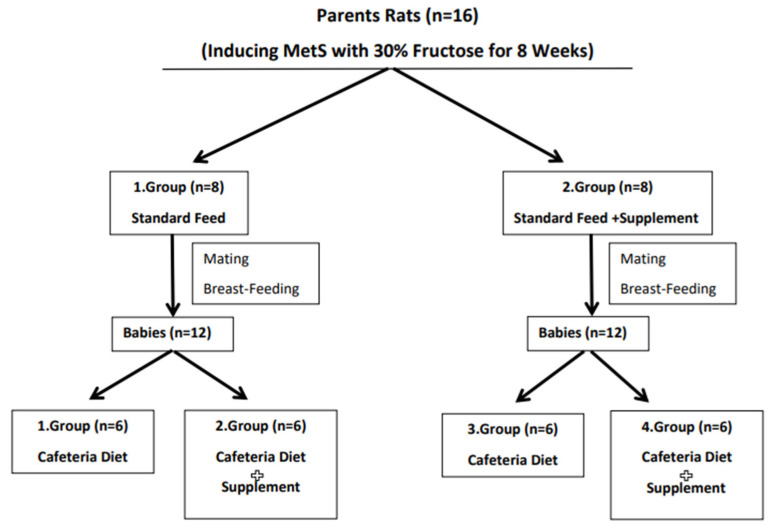
Work plan.

The Wistar Albino parent rats included in the study were randomly divided into 2 different groups, with n = 6 females and n = 2 males in each group, with males and females in different cages. Both groups were fed with 30% fructose solution added to their drinking water and standard feed for MetS induction. After the 8-week intervention period, blood samples were collected from parent rats and MetS parameters (serum fasting glucose, urea, creatine, AST, ALT, ALP, HDL, triglyceride and total cholesterol) were evaluated and compared with reference ranges, and MetS was diagnosed [15]. Then, the control group was named Group 1 and the intervention group was Group 2. Group 1 was fed standard chow and water for eight weeks, while Group 2 was fed standard chow, water and vitamin D supplementation by oral gavage once a week with Devit-3 oral drops (Deva, Tekirdağ, Türkiye) containing 50.000 IU/15 mL vitamin D at a dose of 0.3 mL (1.000 IU/week) per rat [16,17]. After the eight-week intervention, blood samples were collected from the parent rats again. Then, male and female rats in Groups 1 and 2 were mated with each other, and rat pups were born. After the pups were weaned, the parent rats were euthanized. Group 1 offspring and Group 2 offspring were formed by randomly selecting 12 offspring rats from 15 offspring rats born from Group 1, and Group 3 and Group 4 offspring rat groups were formed by randomly selecting 12 offspring rats from 18 offspring rats born from Group 2. Then, initial blood samples were collected from the offspring rats and the intervention was started. During the intervention period (8 weeks), Group 1 pups and Group 3 pups were fed cafeteria diet and water, and Group 2 pups and Group 4 pups were fed cafeteria diet, water and vitamin D supplementation with Devit-3 (Deva, Tekirdağ, Türkiye) oral drops containing 50.000 IU/15 mL vitamin D by oral gavage once a week on the same day and 0.3 mL (1.000 IU/week) vitamin D supplementation once a week. In addition, the control and intervention groups were given standard feed and cafeteria diet containing the same amount of food per animal (20 g/day) throughout the study. After the eight-week intervention period, the rat pups were euthanized and blood samples were collected again. In addition, weekly body weight measurements were taken and recorded throughout the eight-week intervention period. The study plan is shown in Figure 1.

The study was conducted under constant laboratory conditions and the experimental animals were housed under a 12 h light/12 h dark cycle (ambient temperature and humidity were 22–24 °C and 55–60%, respectively).

### 2.6. Collection of Blood Samples

The first blood collection procedure was performed from parent rats for the detection of MetS, and from the offspring rats, after they reached adulthood, under anesthesia, by reaching the orbital sinus with the help of a capillary tube from the medial canthus of the eye, and 0.6 mL of blood was taken per rat. The blood samples were transferred to yellowcapped gel tubes (SST Tubes, BD Microtainer, Wayne, NJ, USA). After sacrifice, blood was collected using 10 mL syringes for maximum cardiac blood collection. Blood taken from the hearts of parent and pup rats was placed in yellow-capped gel biochemistry tubes (Medipax, İstanbul, Türkiye) in the same way. All blood samples were stored at +4 °C by turning then upside down to prevent hemolysis and clotting and centrifuged at 3000× *g* rpm for 25 min on the same day. Then, the serum remaining on the tubes was drawn with an automatic pipette and transferred to 2 mL sterile skirted Eppendorf tubes and stored at −80 °C for biochemical analyses.

### 2.7. Body Weight Measurement (BW)

The body weights of the parent and offspring rats were measured with a scale sensitive to 0.1 g at the same time every day, once a week, throughout the 8-week intervention period. The first measurement (BW1) was made in week 1 of the intervention and the last measurement (BW8) was made in the last week of the intervention.

### 2.8. Preparing the Cafeteria Diet

There are many different recipes and contents of the cafeteria diet in the literature [18,19,20]. In this study, the content and nutrient composition of the cafeteria diet based on the data in the literature are as follows. Assuming that the rats consumed an average of 20 g of dry feed per day, a cafeteria diet consisting of 50 g of salted peanuts, 50 g of cheddar cheese, 50 g of jelly biscuits, 50 g of sausage, 50 g of cheese biscuits, 75 g of milk chocolate, 50 g of potato chips, 75 g of cocoa biscuits and 50 g of granulated sugar was prepared by mixing freshly in a mixer, dried in the oven to the consistency of pellet feed and given to the rats. The daily energy value was 2300 calories in total and an average of 96 calories per rat. The carbohydrate content of the diet was 235 g, 41% of energy, the protein content was 51 g, 9% of energy and the fat content was 128 g, 50% of energy. In the cafeteria diet, 40% of the energy from fats came from saturated fats and 20% of the energy from carbohydrates came from simple sugars. The salt content of the cafeteria diet was 2% (10 g) and the fiber content was 4% (20 g). The rest consisted of water, vitamins, minerals and ash. The standard rat diet contained 24% protein, 58% carbohydrate, 11% fat, 6% fiber and 1% salt, vitamins, minerals and ash.

### 2.9. Application of Euthanasia

Injectable anesthetics were used for euthanasia. Briefly, 2% xylazine (Xylazinbo, Intermed, Ankara, Türkiye) was used as a premedicant, and ketamine (Ketasol, İnterhas, Ankara, Türkiye) from the dissociative group was used as an anesthetic. The dosages of ketamine and xylazine were determined as 10 mg/kg and 80 mg/kg, respectively. After the injection, the effect of anesthesia was checked by applying pressure to the tail and observing eyelid reflexes, and cervical dislocation was applied according to the effect of the anesthetic. Then, blood samples were taken.

### 2.10. Ethical Approval

To start the study, ethical approval was obtained from the Erciyes University Animal Experiments Local Ethics Committee (EÜHADYEK), dated 5 April 2023 and numbered 23/065.

### 2.11. Statistical Analysis

Statistical analyses of the study were performed using the SPSS 27.0 (IBM Inc, Armonk, NY, USA) program. The conformity of continuous numerical measurements to normal distribution was analyzed using the Shapiro–Wilk method. Descriptive measurements were presented as mean ± SD. Student’s t-test was used for comparisons of two independent groups, and a One-Way Analysis of Variance was used for comparisons made according to multiple groups. The homogeneity of variances was checked with the Levene test. Since variances of all measurements were found to be homogeneous, the Tukey HSD post hoc test was preferred for pairwise comparisons. Cohen’s D estimated values and eta-squared correlation value were also given, together with the analysis results, as the effect size for the analyses. In all analyses, the *p* < 0.05 value for a type-I error value of 5% was considered statistically significant.

## 3. Results

This study was designed as an experimental animal study. During this study (8 weeks = 56 days), parent rats consumed a total of approximately 9 kg of standard chow in Group 1 and Group 2. In the offspring rats, Group 1, Group 2, Group 3 and Group 4 consumed a total of approximately 27 kg of cafeteria diet, with an average of 6.7 kg, respectively. There was no difference in the amount of food consumption in both parent rats and offspring. Therefore, there was no relationship between vitamin D intake and food consumption. First, the measured values for the eight rats on the normal diet and normal diet plus supplementary diet were compared between the study groups. All biochemistry measurements of the parent rats before and after the intervention were compared between the diet groups, and it was observed that the post-intervention measurements generally showed significant differences between the groups. Urea, glucose and creatine values were significantly lower in the supplemented diet (*p* < 0.001). ALT, AST and ALP liver enzymes were significantly lower in the supplement group (*p* < 0.001 and *p* = 0.002). Post-HDL and -TG values did not differ significantly between the groups, whereas total cholesterol values showed a significant decrease (*p* < 0.001). Body weights of the rats were measured in eight different periods, but the weights were similar between the groups (*p* > 0.05) (Table 1).

Four different diet groups were created for the offspring of mothers who came from two different diet groups. Pre- and post-intervention biochemical values of the offspring were determined and compared between the groups. Post-urea value was found to be significantly higher in the cafeteria diet group (*p* < 0.001). Pre-glucose measurements were significantly higher in the cafeteria and cafeteria and supplemental diet groups, and lower in the groups where mothers were given a diet (*p* = 0.005). Post-intervention glucose values were significantly lower in the supplemental diet mother and offspring group (*p* < 0.001).

Post-creatine value was significantly higher in the cafeteria-only diet group, while it was significantly lower in the cafeteria + supplementation diet group (*p* = 0.034). Pre-ALT value increased in the group where mothers and pups received a supplemental diet (*p* = 0.005). Post-ALT value was significantly higher in the group of mothers receiving a normal diet, while it was lower in the groups that were given a supplementary diet (*p* < 0.001). Pre-AST values did not show a significant difference, while post-AST value was significantly lower in the group in which mothers and offspring were given a supplementary diet (*p* < 0.001).

Pre-ALP values were higher in the supplemented groups (*p* = 0.001), while post-ALP values were lower in the mother and offspring supplemented group (*p* < 0.001). Both pre- and post-HDL values were lower in the cafeteria diet group, while they were significantly higher in the mother and offspring-supplemented group (*p* = 0.002 and *p* < 0.001, respectively). Triglyceride and total cholesterol measurements did not show any significant differences between the groups before the intervention but were significantly lower in the mother and offspring supplemented group after the procedure (*p* < 0.001).

Of the body weight values measured in eight different periods, all measurements except the second measurement showed significant differences between the groups (*p* < 0.05). In general, body weight was found to be significantly lower in the mother and the cafeteria + supplementation diet group compared to the other groups (Table 2).

## 4. Discussion

MetS is a complex disease that is affected by environmental factors that have a strong relationship with nutrition, and it can affect future generations and causes damage to epigenetic mechanisms and pathways. One of the methods to cope with this complex disease is vitamin D supplementation [21]. A significant improvement in MetS parameters was found in female rats after two months of vitamin D supplementation by Hoseini et al. [22]. A cohort study by Lee et al. [23] on 2936 people found a positive correlation between low vitamin D levels and waist circumference, LDL cholesterol levels, and triglyceride levels in participants. In this study, similarly, in the control group of parent rats, serum urea, glucose, creatine, total cholesterol, ALP, AST and ALT levels were found to be significantly higher than in the intervention group, without any significant difference in body weight (*p* < 0.05) (See Table 1).

These differences confirm the development of MetS caused by high fructose and demonstrate the ameliorative effect of vitamin D on MetS parameters. Similar to studies in the literature, the main mechanism of this effect is thought to be due to the stimulation of pancreatic beta cells by vitamin D, regulating apolipoprotein A-1 levels and playing an important role in cholesterol transport and reducing tissue damage by controlling inflammatory processes [24,25].

In the literature, epigenetic aging is the leading cause of MetS development. The leading causes of epigenetic aging are increased glycemic load, obesity, increased saturated fat consumption and dyslipidemia. On the other hand, adequate vitamin D consumption is known to delay epigenetic aging and positively affect MetS parameters [26,27,28]. Therefore, diet is one of the most important environmental factors that can affect epigenetic mechanisms (histone modification, DNA methylation, miRNA expression, etc.). High fat and high simple carbohydrate diets are known to negatively affect MetS parameters [29].

In a study conducted by Palma-Jocinto et al. [30], plasma total cholesterol, HDL and LDL cholesterol, triglyceride, glucose and insulin profiles of male rats fed with a cafeteria diet for 38 weeks altered. Serum insulin, fasting glucose, LDL cholesterol, total cholesterol, TNF-α, IL-6 and triglyceride levels were abnormally elevated. In addition, serum HDL levels decreased. In an experimental study conducted with mice by Zheng et al. [31], it was found that feeding mice with a high-calorie diet causes impaired glucose intolerance and may cause impairments in miRNA expression.

A systematic review study by Mansour-Gahanaei et al. [32] found that daily vitamin D supplementation of 3.000 IU or more had an ameliorative effect on liver enzymes (ALT and AST). A meta-analysis by Tabrizi et al. [33] found that vitamin D supplementation did not have much effect on liver enzymes in patients with fatty liver disease. In this study, when the pre-intervention MetS data of the pups born to mothers with MetS were compared, it was found that the serum glucose and body weights of the pups of the supplemented parents were significantly lower, and ALT, ALP and HDL levels were significantly higher, compared to the pups of the non-supplemented mothers (*p* < 0.05). It was observed that the creatine level was higher in Group 4 than in Group 2 after the intervention in the offspring groups. This difference was not significant. However, this difference was found to be significant in Group 3 and Group 4 from the same parents. The fact that ALP levels were higher in Group 3 and Group 4 before the intervention compared to the control groups suggests that impaired epigenetic mechanisms from parents after MetS cannot be fully repaired by vitamin D for some MetS parameters. Although vitamin D had a favorable effect on many MetS parameters, it did not have much effect on creatine, ALT and AST. However, epigenetic studies are needed to fully clarify this situation. Furthermore, when MetS parameters were compared after cafeteria diet and vitamin D supplementation, it was found that the cafeteria diet increased MetS parameters and posed a risk for MetS development. In addition, serum urea, glucose, creatinine, ALT, AST, ALP, triglyceride, total cholesterol, triglyceride and total cholesterol levels and body weights of the supplementation groups were significantly lower, while serum HDL levels were significantly higher (*p* < 0.05). In addition, it was found that the cafeteria diet caused an increase in body weight in all groups with and without vitamin D. However, this effect was seen more in the groups without vitamin D (see Table 2).

According to the data in the literature, the main source of this change in MetS parameters is that vitamin D supplementation affects the epigenetic mechanisms for some MetS parameters [34,35,36]. This study also shows that even if there is an environmental effect caused by a poor diet such as the cafeteria diet, vitamin D supplementation can improve some MetS parameters by affecting epigenetic mechanisms. However, more studies are needed to fully explain the underlying mechanism.

## 5. Conclusions

In conclusion, diet is a crucial environmental factor to prevent and/or manage the development of MetS, which is characterized by obesity, dyslipidemia, glucose intolerance and impaired liver enzymes. In this study, we found that weekly vitamin D supplementation of 0.3 mL (1.000 IU/week) administered by oral gavage to parent and offspring rats had a positive effect by reducing some MetS parameters in a dietary model consisting of high fat and high simple carbohydrates. However, no significant results were found regarding the genetic transmission of MetS. In addition, more studies are needed to determine the effect of vitamin D on genetic and/or epigenetic mechanisms.

## 6. Limitations of the Study

One of the limitations of this study is that the body weight measurements of the parent and offspring rats began after the first week of the intervention, not before the intervention, so no determination could be made regarding the initial weights.

## Figures and Tables

**Table 1:** Pre- and post-intervention measurements of mothers according to study groups.

	Group 1 (Parent) Normal Diet	Group 2 (Parent) Normal Diet ± Supplement		Cohen’s D	
Measurements	Mean ± SD		*p*	Point Estimation	Standardized Value
Urea pre- (mg/dL)	86.75 ± 4.06	89.50 ± 2.56	0.128	−0.810	3.40
Urea post- (mg/dL)	65.00 ± 4.75	54.00 ± 4.84	<0.001 *	2.294	4.80
Glucose pre- (mg/dL)	304.80 ± 21.70	305.63 ± 22.0	0.941	−0.038	21.85
Glucose post- (mg/dL)	288.89 ± 20.58	246.36 ± 5.90	<0.001 *	2.809	15.14
Creatine pre- (mg/dL)	1.62 ± 0.07	1.61 ± 0.04	0.742	0.168	0.06
Creatine post- (mg/dL)	1.56 ± 0.05	1.42 ± 0.04	<0.001 *	3.211	0.05
ALT pre- (U/L)	112.13 ± 7.42	105.40 ± 11.00	0.174	0.717	9.38
ALT post- (U/L)	105.28 ± 5.19	78.84 ± 6.42	<0.001 *	4.531	5.83
AST pre- (U/L)	306.21 ± 36.63	300.73 ± 25.01	0.732	0.175	31.37
AST post- (U/L)	289.95 ± 34.08	235.01 ± 23.57	0.002*	1.874	29.31
ALP pre- (U/L)	299.50 ± 15.52	303.0 ± 22.55	0.723	−0.181	19.30
ALP post- (U/L)	275.38 ± 15.64	208.5 ± 26.48	<0.001 *	3.075	21.75
HDL pre- (mg/dL)	20.5 ± 1.20	20.0 ± 2.27	0.590	0.276	1.81
HDL post- (mg/dL)	22.0 ± 1.51	23.0 ± 1.77	0.245	−0.607	1.65
TG pre- (mg/dL)	73.5 ± 14.77	92.5 ± 4.40	0.008 *	−1.743	10.90
TG post- (mg/dL)	69.0 ± 15.19	70.5 ± 4.17	0.795	−0.135	11.14
TOC pre- (mg/dL)	177.0 ± 18.24	181.75 ± 35.24	0.740	−0.169	28.06
TOC post- (mg/dL)	164.25 ± 12.76	125.75 ± 17.04	<0.001 *	2.557	15.06
BW1 (g)	217.50 ± 71.07	225.38 ± 73.95	0.831	−0.109	72.53
BW2 (g)	240.88 ± 70.79	258.25 ± 65.62	0.619	−0.255	68.26
BW3 (g)	253.50 ± 73.90	269.13 ± 64.40	0.659	−0.225	69.32
BW4 (g)	267.81 ± 71.70	278.88 ± 63.75	0.749	−0.163	67.84
BW5 (g)	284.06 ± 69.75	291.25 ± 61.75	0.830	−0.109	65.87
BW6 (g)	291.38 ± 68.11	297.94 ± 60.73	0.842	−0.102	64.53
BW7 (g)	288.25 ± 67.44	296.50 ± 59.99	0.800	−0.129	63.83
BW8 (g)	282.75 ± 66.93	291.44 ± 59.91	0.788	−0.137	63.52

*: Significant at 0.05 level according to Student’s *t*-test; ALT, alanine aminotransferase; AST, aspartate transferase; ALP, alkaline phosphatase; TG, triglyceride; TOC, total cholesterol; BW, body weight.

**Table 2:** Pre- and post-intervention measurement values of the offspring according to the study groups.

	Group 1 Offspring	Group 2 Offspring	Group 3 Offspring	Group 4 Offspring		Eta Square
Measurements	Mean ± SD				*p*	
Urea pre- (mg/dL)	81.90 ± 4.35	79.18 ± 4.42	79.62 ± 4.076	79.88 ± 2.14	0.632	0.081
Urea post- (mg/dL)	97.05 ± 6.61	81.81 ± 4.87	85.35 ± 2.80	81.38 ± 2.77	<0.001 *	0.699
Glucose pre- (mg/dL)	106.52 ± 8.19	103.12 ± 6.48	92.43 ± 7.22	92.62 ± 7.38	0.005 *	0.466
Glucose post- (mg/dL)	128.73 ± 9.62	112.63 ± 6.96	129.75 ± 4.67	105.41 ± 3.73	<0.001 *	0.747
Creatine pre- (mg/dL)	1.15 ± 9.62	1.15 ± 6.96	1.13 ± 4.67	1.17 ± 3.73	0.975	0.010
Creatine post- (mg/dL)	1.37 ± 0.11	1.17 ± 0.14	1.32 ± 0.12	1.22 ± 0.14	0.034 *	0.346
ALT pre- (U/L)	54.25 ± 6.02	52.32 ± 4.12	59.27 ± 1.87	60.73 ± 3.17	0.005 *	0.464
ALT post- (U/L)	79.08 ± 4.48	64.40 ± 4.27	82.68 ± 3.08	63.55 ± 2.88	<0.001 *	0.862
AST pre- (U/L)	88.65 ± 5.78	89.97 ± 6.65	91.13 ± 2.39	88.33 ± 3.63	0.746	0.058
AST post- (U/L)	119.08 ± 7.03	102.37 ± 8.68	124.88 ± 7.29	96.55 ± 2.96	<0.001 *	0.574
ALP pre- (U/L)	164.33 ± 5.24	161.67 ± 4.63	172.33 ± 4.93	172.33 ± 2.73	0.001 *	0.910
ALP post- (U/L)	217.67 ± 13.14	182.00 ± 9.34	271.17 ± 17.22	190.33 ± 4.76	<0.001 *	0.511
HDL pre- (mg/dL)	25.67 ± 2.73	25.67 ± 3.72	30.33 ± 2.94	31.83 ± 2.23	0.002 *	0.742
HDL post- (mg/dL)	21.50 ± 2.67	24.17 ± 2.86	23.50 ± 1.05	30.83 ± 2.04	<0.001 *	0.049
TG pre- (mg/dL)	20.83 ± 1.72	21.42 ± 2.37	21.67 ± 0.82	21.00 ± 0.89	0.792	0.730
TG post- (mg/dL)	33.17 ± 3.31	26.67 ± 1.97	28.50 ± 2.074	23.83 ± 1.17	<0.001 *	0.146
TOC pre- (mg/dL)	58.00 ± 3.85	57.83 ± 3.92	55.17 ± 1.60	57.67 ± 2.25	0.357	0.920
TOC post- (mg/dL)	77.33 ± 3.56	61.83 ± 3.06	81.33 ± 2.07	61.83 ± 2.56	<0.001 *	0.476
BW1 (g)	267.33 ± 28.99	218.42 ± 14.61	263.67 ± 34.10	225.75 ± 17.92	0.004 *	0.476
BW2 (g)	279.33 ± 29.52	227.08 ± 16.34	273.58 ± 33.73	247.83 ± 51.2	0.063	0.300
BW3 (g)	290.50 ± 29.79	236.58 ± 18.64	284.83 ± 32.28	237.75 ± 17.80	0.001 *	0.543
BW4 (g)	301.17 ± 30.47	244.33 ± 18.19	294.75 ± 32.42	243.58 ± 17.90	0.001 *	0.543
BW5 (g)	313.42 ± 32.06	253.92 ± 17.39	306.50 ± 35.05	249.08 ± 17.04	<0.001 *	0.593
BW6 (g)	327.58 ± 33.72	263.50 ± 18.60	318.42 ± 34.87	256.42 ± 16.71	<0.001 *	0.620
BW7 (g)	339.33 ± 33.71	272.42 ± 18.42	329.42 ± 33.01	262.25 ± 16.45	<0.001 *	0.660
BW8 (g)	354.75 ± 32.51	286.25 ± 16.43	342.83 ± 33.47	267.58 ± 15.61	<0.001 *	0.707

*: Significant at 0.05 level according to one-way ANOVA test; ALT, alanine aminotransferase; AST, aspartate transferase; ALP, alkaline phosphatase; TG, triglyceride; TOC, total cholesterol; BW, body weight.

## Data Availability

The data from this study can be used by other authors upon request.
